# Nonviral mcDNA-mediated bispecific CAR T cells kill tumor cells in an experimental mouse model of hepatocellular carcinoma

**DOI:** 10.1186/s12885-022-09861-1

**Published:** 2022-07-25

**Authors:** Hezhi Wang, Xiaoxiao Wang, Xueshuai Ye, Yi Ju, Nana Cao, Shuqi Wang, Jianhui Cai

**Affiliations:** 1 Department of Liver Surgery and Transplantation, Liver Cancer Institute, Zhongshan Hospital, Fudan University, Shanghai, 200032 China; 2 Key Laboratory of Carcinogenesis and Cancer Invasion, Ministry of Education, Fudan University, Shanghai, 200032 China; 3 Department of Endocrinology, People’s Hospital of Longhua, Shenzhen, 518109 China; 4 Department of Surgery, Hebei Medical University, Shijiazhuang, 050000 China; 5 Department of Medicine, Medical College of Hebei University of Engineering, Handan, 056002 China; 6 College of Life Sciences, Hebei Normal University, Shijiazhuang, 050000 China; 7 Department of Anorectal Surgery, Shijiazhuang Traditional Chinese Medicine Hospital, Shijiazhuang, 050000 China; 8 Department of Surgery & Oncology, Hebei General Hospital, Shijiazhuang, 050000 China; 9 Hebei Medical University, 361 East Zhongshan Road, Shijiazhuang, 050017 Hebei China

**Keywords:** Hepatocellular carcinoma, Cancer immunotherapy, Non-viral mcDNA vector, Bispecific CAR T cells, Cancer stem cells

## Abstract

**Background:**

Hepatocellular carcinoma (HCC) is one of the most common cancers worldwide and the adoptive immunotherapy of which is worth studying. CD133, a kind of cancer stem cell (CSC) antigen, together with glypican-3 (GPC3) has been proved to be highly expressed in HCC cells and both of them are used as targets to generate chimeric antigen receptor (CAR) T cells. But there are limitations like “off-target” toxicity, low transfection efficacy and weak antitumor ability in CAR T cells treatment.

**Methods:**

The peripheral blood was acquired from healthy donors and T cells were separated by density-gradient centrifugation. We used an electroporation system to deliver anti-CD133 and anti-GPC3 single chain Fragment variable (scFv) structures as target genes into the T cells. The cell membrane was opened by the momentary electric current effect, and the target gene was delivered into the cell by non-viral minicircle DNA (mcDNA) vector. The flow cytometry and western blot assays were used to detect whether the two scFv were simultaneously transfected and the transfection efficacy of this bispecific CAR T cell generation method. We respectively detected the in vitro and in vivo tumor-suppression efficacy of CAR T cells through the CCK-8 assays and the HCC xenograft mice models. The CoG133-CAR T cells containing both CD133 and GPC3 antigen recognition sites were the effector cells. CD133-CAR T cells and GPC3-CAR T cells were defined as single-targeted control groups, normal T and mock T cells were defined as blank control groups.

**Results:**

The mcDNA vector accommodated two target gene structures successfully transfected to generate bispecific CAR T cells. The detection methods on gene level and protein level confirmed that CoG133-CAR T cells had considerable transfection efficiency and exhibited both antigen-binding capacity of CD133 and GPC3. Compared to single-targeted CAR T cells or control T cells, CoG133-CAR T cells performed enhanced eliminated efficacy against CD133 and GPC3 double-positive HCC cell line in vitro and HCC xenograft mice in vivo. Hematoxylin and eosin (H&E) staining indicated no fatal “off-target” combination existed on CoG133-CAR T cells and major organs.

**Conclusion:**

Our study suggests that it is with higher efficiency and more safety to prepare bispecific CAR T cells through non-viral mcDNA vectors. CoG133-CAR T cells have enhanced tumor-suppression capacity through dual antigen recognition and internal activation. It provides an innovative strategy for CAR T therapy of HCC, even solid tumors.

**Supplementary Information:**

The online version contains supplementary material available at 10.1186/s12885-022-09861-1.

## Background

Primary liver cancer is the sixth most common cancer and the second leading cause of cancer mortality worldwide [1]. Hepatocellular carcinoma (HCC) is the most common type of primary liver cancer, and more than 80% of cases are associated with the most common risk factor of liver cirrhosis, which resulting predominantly from chronic hepatitis B virus (HBV) or hepatitis C virus (HCV) infection and alcoholic liver disease [2]. Since the overall 5-year survival rate of HCC patients is less than 16%, the development of innovative treatments for HCC is urgently needed [3, 4].

Traditional therapeutic methods, including chemical drugs, radiotherapy, ablation, transcatheter arterial chemoembolization (TACE), and surgery, seldom achieve satisfactory effects [5]. These methods are limited by substantial induced suffering, massive cost, iatrogenic metastasis risk and poor prognosis. Immunotherapy with chimeric antigen receptor (CAR)-engineered T cells, which mobilizes internal immunocytes to achieve efficient and painless antitumor outcomes, has been continuously explored and improved. CAR T cells first achieved clinical remission (CR) in a B-cell precursor acute lymphoblastic leukemia patient treated with CD19-CAR T cells, and CD19-CAR T cells were approved as a commercial product by the Food and Drug Administration (FDA) for clinical therapy in 2017 [6, 7]. CAR T cell therapy has been nominated by the American Society of Clinical Oncology (ASCO) as the most important advancement in cancer research [8], and its therapeutic efficacy has been proven effective in solid tumors [9, 10]. We demonstrated that the 3rd-generation CAR T cells produced by our platform, including PSCA-CAR T cells against prostate cancer and NKG2D-CAR T cells against colorectal cancer, possess significant antitumor capacity both in vitro and in vivo [11, 12].

CAR T cells have three components: 1) an extracellular single-chain variable fragment (scFv), which can specifically bind tumor-associated antigens (TAAs) through human leukocyte antigen (HLA)-independent recognition; 2) a hinge domain and transmembrane fragment from human CD8α; and 3) at least one intracellular costimulatory domain such as that from human CD28, CD137 or CD3ζ to promote cell proliferation and the release of cytokines and cytotoxic granules after activation by targeted tumor signals. The method of introducing target sequences into T cells via virus-derived vectors is extensively used. However, this method is often subject to limitations such as safety concerns, low transfection efficacy and considerable cost [13–15]. In addition, the limitations of single antigen-specific CAR T cell treatment, such as “off-target” toxicity and narrow targetability, are difficult to eliminate [16, 17]. To overcome the abovementioned obstacles, we designed CAR T cells based on the progress already achieved by our research team. The newly designed CAR T cells are modified by a nonviral minicircle DNA (mcDNA) vector and featured two scFv structures, thus producing an effective, low-cost and safe treatment.

McDNA vectors are free of bacterial DNA and highly expressed in cells [18]. Glypican-3 (GPC3) is a hallmark of HCC, with the positive expression on 75% of HCC cells, and CD133 is a kind of cancer stem cell (CSC) maker that is also specifically expressed on HCC cells. Both of these antigens induce significant antitumor function in immunotherapy and have been the subject of widespread clinical trials on CAR-related treatments [19–22]. In this study, we confirmed that simultaneously electroporating two mcDNA vectors containing different target genes is a viable strategy to generate bispecific CAR T cells. With adding cytokines including CD3/CD28 antibodies, IL-2, IL-15 and IFN-γ, CoG133-CAR T cells proliferate to an adequate amount for HCC elimination. The stability of generation strategy is confirmed through gene level and protein level. Both in vitro and in vivo assays to indicate that the suppression efficacy of CoG133-CAR T cells against HCC was stronger than that of single-targeted CAR T cells. In summary, this mcDNA-based bispecific CAR T cell system amplified signaling cascade activity in the cell population and exhibited stronger oncolytic activity in terms of cell quality. Moreover, it provides considerable prospects for the development of a new generation of CAR T cells.

## Materials and methods

### Construction of parental plasmid vectors and production of mcDNA

Based on previous reports, we designed a third-generation GPC3-CAR structure [23] and a second-generation CD133-CAR structure [24, 25]. The DNA sequences of GPC3 scFv and CD133 scFv were derived from monoclonal antibodies (mAbs) described by Nakano [26] and Swaminathan [27]. The GPC3-CAR was composed of the GPC3 scFv, human CD8α hinge and transmembrane domain (nucleotides 412–609, GenBank NM 001,768.6), human CD28 molecule (nucleotides 538–660, GenBank NM 006,139.3), human CD137 molecule (nucleotides 640–765, GenBank NM 001,561.5) and human CD3ζ molecule (nucleotides 154–492, GenBank NM 198,253.2). The CD133-CAR, containing the CD133 scFv, was linked to the intracellular domains from the human CD137 and CD3ζ molecules via the human CD8α hinge and CD8α transmembrane regions. NcoI and EcoRI sites were incorporated at both ends. We humanized the two CAR gene sequences and synthesized them (Detai Biologics, Nanjing, China), and confirmed them by genetic sequencing (Sango Biotech, Shanghai, China). We cloned these two CAR structures into pUC57 vectors and then transformed into the parental minicircle plasmid pMC.CMV-Easy™ (System Biosciences, CA, USA). The pMC.CMV-Easy-GFP-CD133-CAR (8513 bp) parental minicircle plasmid contained the CD133-CAR (1455 bp) and a GFP cassette (758 bp), and pMC.CMV-Easy-GPC3-CAR (7923 bp) contained the GPC3-CAR (1608 bp) without a GFP cassette (to clearly distinguish the constructs in subsequent experiments). We transformed the parental minicircle plasmids into *E. coli* strain ZYCY10P3S2T (System Biosciences), and then added the inducer L- ( +)-arabinose (Sigma Chemical, MO, USA) into the bacterial growth medium to mediate recombination between *att*B and *att*P. The recombinase ΦC31 was produced after the recombination and separated the parental minicircle plasmid into mcDNA and the parental bacterial backbone. We extracted the CD133-CAR mcDNA and GPC3-CAR mcDNA with an Endo-Free Plasmid DNA Maxi Kit (Omega Bio-tek, GA, USA) and confirmed them via restriction analysis.

### Generation and proliferation of CoG133-CAR T cells

Peripheral blood mononuclear cells (PBMCs) derived from healthy donors were obtained from the Hebei Blood Center. All donors gave informed consent to use their samples for research purposes. All procedures were performed in accordance with the guidelines approved by Hebei Medical University. PBMCs were isolated with lymphocyte separation medium (Tonbo Biosciences, CA, USA). Primary human CD3^+^ T cells were positively selected from PBMCs with MACS CD3 MicroBeads (Miltenyi Biotec, Bergish Gladbach, Germany) and cultured in RPMI-1640 medium (Thermo Fisher Scientific, MA, USA) supplemented with 10% heat-inactivated fetal bovine serum (FBS, Thermo Fisher Scientific) at 37 °C in 5% CO_2_. Primary T cells were activated with 1000 U/L IFN-γ (Peprotech, NJ, USA), cultured with 1 μg/ml anti-CD3 and anti-CD28 antibodies (Miltenyi Biotec) for 1 day, and then expanded in the presence of 500 U/ml recombinant human interleukin-2 (IL-2, Peprotech) and 10 U/ml recombinant human interleukin-15 (IL-15, Peprotech) for 2–5 days. We transfected 5 × 10^6^ T cells via electroporation with a 4D-Nucleofector™ system (Lonza, Cologne, Germany); 3 μg of mcDNA control plasmid (System Biosciences), CD133-CAR plasmid or GPC3-CAR plasmid, and 100 μl of P3 Primary Cell Buffer (Lonza) was added according to the manufacturer’s instructions. The EO-115 program was used. CoG133-CAR T cells were generated by simultaneously electroporating 1.5 μg of CD133-CAR plasmid and 1.5 μg of GPC3-CAR plasmid into T cells. The transfected T cells were cultured in fresh medium supplemented with 500U/ml IL-2. Fresh medium was added every other day to maintain a concentration of 8 × 10^5^cells/ml.

### Cell lines and culture conditions

The human HCC cell lines HepG2 and PLC8024 were obtained from the American Type Culture Collection (ATCC, VA, USA) and cultured in minimal essential medium (MEM, Thermo Fisher Scientific). Huh7 and SK-HEP-1 cells were obtained from the Shanghai Cell Bank (Shanghai, China) and cultured in Dulbecco’s modified Eagle’s medium (DMEM, Thermo Fisher Scientific). All cell lines were cultured in medium supplemented with 10% FBS (Thermo Fisher Scientific) and 1% penicillin–streptomycin (Thermo Fisher Scientific) at 37 °C in 5% CO_2_. For bioluminescence assays, we generated a firefly luciferase expressing Huh7 cell line.

### Flow cytometry

All cell samples were analyzed with a BD FACSCanto™ flow cytometry system (BD Bioscience, CA, USA), and statistical analysis was conducted in FlowJo software (FlowJo, OR, USA). The phenotype of T cells was assessed with fluorescently labeled antibodies specific for human CD3-PC5, CD4-fluorescein isothiocyanate (FITC) and CD8-FITC, which were obtained from BD Bioscience. Tumor surface antigen expression was detected with antibodies against human CD133-phycoerythrin (PE) (BioLegend, CA, USA) and GPC3-PE (Abcam, MA, USA); isotype control groups were stained with IgG1-PE (Abcam). The expression of GFP in T cells was evaluated FL1 channel to demonstrate the expression of CD133-CAR. The expression of GPC3-CAR was assessed by recombinant biotinylated protein L (Thermo Fisher Scientific) binding PE-conjugated streptavidin (PE-SA, BD Bioscience). All FACS-related cell samples were handled on ice and washed three times with 1 × PBS (Thermo Fisher Scientific) containing 1% FBS before staining the corresponding antibodies.

### In vitro cytotoxicity assays

Effector cells were cocultured with target cells at increasing effector: target ratios of 1:5, 1:1, 5:1 and 10:1 in flat-bottom 96-well plates (Corning, NY, USA) containing 100 μl of T cell culture medium at 37 °C in 5% CO_2_ for 18 h. Then, we measured the absorbance at 450 nm according to the Cell Counting Kit-8 instructions (Dojindo Molecular Technologies, Kumamoto, Japan) using an Epoch microplate spectrophotometer (BioTek, VT, USA). We calculated the cytotoxicity of the effector cells with the following formula: specific lysis (%) = [1– (mixture cell experiment–medium control)/ (target cell spontaneous–medium control)] × 100.

### Cytokine secretion assays

Effector cells were cocultured with target cells in 96-well plates at an effector: target ratio of 5:1 for 24 h. Supernatants were collected to measure the levels of cytokines, including IL-2, IFN-γ and TNF-α, according to the protocols of the enzyme-linked immunosorbent assay (ELISA) kit (Thermo Fisher Scientific). Additionally, 5 × 10^6^ effector cells were collected for in vitro experiments, and 100 μl of peripheral blood was collected from treated xenograft mice for in vivo experiments.

### Western blot analysis

T cells and tumor tissues were lysed with Radioimmunoprecipitation (RIPA) Lysis and Extraction Buffer (Thermo Fisher Scientific) and quantified with a BCA Protein Assay Kit (Thermo Fisher Scientific). Protein lysates were separated on a 12% sodium dodecyl sulfate–polyacrylamide gel electrophoresis (SDS-PAGE) gel and transferred to a polyvinylidene fluoride (PVDF) membrane (Thermo Fisher Scientific). The PVDF membrane was blocked in AquaBlock Blocking Buffer (EastCoast Bio, ME, USA) for 2 h, followed by overnight incubation at 4 °C with the following primary antibodies: anti-CD133 (1:1000, Abcam), anti-GPC3 (1:400, Abcam), anti-β-actin (1:5000, Abcam) and anti-CD3ζ (1:5000, Abcam). Unbound antibodies were washed away with Tris–HCl buffer containing Tween 20, and the PVDF membrane was then incubated with a horseradish peroxidase (HRP)-conjugated secondary antibody (Abcam) for 50 min at room temperature. Blots were detected using SuperSignal™ West Pico PLUS Chemiluminescent Substrate (Thermo Fisher Scientific) and visualized with a ChemiDoc™ Touch Imaging System (BIO-RAD, CA, USA).

### Xenograft mouse models

All animal experiments were conducted in the Clinical Research Center of Hebei General Hospital (HBGH), and all animal procedures were approved by the Animal Care and Management Committee of HBGH. All animal protocols were approved by the Hebei Medical University Animal Care and Use Committee, Hebei, China. Six- to eight-week-old female non-obese diabetic/severe combined immuno-deficiency (NOD/SCID) mice were purchased from Vital River, Beijing, China and were raised in specific pathogen-free (SPF)-grade cages and provided autoclaved food and water.

For the subcutaneous HCC models, mice were inoculated subcutaneously with 5 × 10^6^ SK-HEP-1, HepG2, PLC8024 or Huh7 cells on day 0, and the volumes of tumors derived from these cells were 100mm^3^ on day 14, day 12, day 17 and day 15, respectively. Then, the xenograft mice received two intravenous injections of 1 × 10^7^ effector cells on the 3rd and 10th days after the tumor volume reached 100 mm^3^. For the bioluminescent Huh7 models, mice received 5 × 10^6^ luciferase-labeled Huh7 cells subcutaneously and were then divided randomly into 5 groups (*n* = 5) and injected intravenously with two doses of 1 × 10^7^ effector cells at the abovementioned time points. We measured the tumor volumes and mouse body weights three times weekly, and tumor volumes were calculated with the following formula: V = 1/2 (length × width^2^). Tumor weights were measured after the mice were sacrificed.

### Histopathological, immunohistochemical and immunofluorescence analyses

After sacrifice, the Huh7 xenograft mice were perfused with saline and paraformaldehyde at the apex of the heart, and the heart, liver, brain, lung, pancreas, spleen, and intestine were placed in a paraformaldehyde fixative for more than 24 h. All tissues were embedded in paraffin and sliced. Paraffin sections were first dewaxed and stained with hematoxylin. Then, the sections were dehydrated in an alcohol gradient and stained with eosin. Finally, the sections were sealed in neutral gum after dehydration. Three sections were randomly selected from each mouse and photographed under an optical microscope (NIKON, Tokyo, Japan). Paraffin sections of mouse tumors were subjected to the HE staining method described above. After dewaxing, the tumor tissue sections were placed in a repair kit filled with EDTA antigen retrieval buffer (pH 8.0) for repair. A tissue pen was used to outline the tissue, and an autofluorescence quencher was added. Bovine serum albumin (BSA) was added dropwise in the circle for 30 min. For immunohistochemical staining, sections were incubated with anti-CD133 (1:1000, Abcam) and anti-GPC3 (1:200, Abcam) antibodies at 4 °C overnight and were then washed and incubated with the corresponding secondary antibody at room temperature for 50 min. Color development was carried out with 3,3’-diaminobenzidine (DAB), and nuclei were counterstained with hematoxylin. Finally, sections were observed and images were acquired under a microscope. For immunofluorescence staining, sections were incubated with anti-CD133 (1:1000, Abcam), anti-GPC3 (1:200, Abcam) and anti-CD3ζ (1:200, Abcam) antibodies overnight at 4 °C and then with the corresponding secondary antibody for 50 min at room temperature after washing. After incubation with 4’,6-diamidino-2-phenylindole (DAPI) for 10 min at room temperature, images were acquired under a fluorescence microscope (NIKON, Tokyo, Japan).

### Bioluminescence assays

Cultured Huh7 cells were inoculated bilaterally into the backs of mice to observe tumor growth, and tumors were imaged in vivo when the average volume reached 100mm^3^. Ten minutes after subcutaneous injection of 100 mg/kg D-fluorescein (Solarbio, Beijing, China), mice were anesthetized with isoflurane and were then imaged with a cooled charge-coupled device (CCD) camera system (IVIS Lumina LT Series III, Perkin Elmer, Waltham, MA, USA). The results were analyzed quantitatively in Living Image software.

### Statistical analysis

Data are presented as the means ± SDs and were analyzed using Prism 8.0 (GraphPad Software, San Diego, CA). Statistical analysis was carried out using Student’s t-test (two-group comparisons), one-way ANOVA with Tukey’s post hoc test, and two-way repeated-measures ANOVA followed by Bonferroni’s post hoc test. Comparison of survival curves was performed using the log-rank (Mantel-Cox) test. *P* < 0.05 was considered statistically significant.

## Results

### Construction of the nonviral mcDNA vector and generation of GPC3 and CD133 bispecific CAR-engineered T cells

**Fig. 1:**
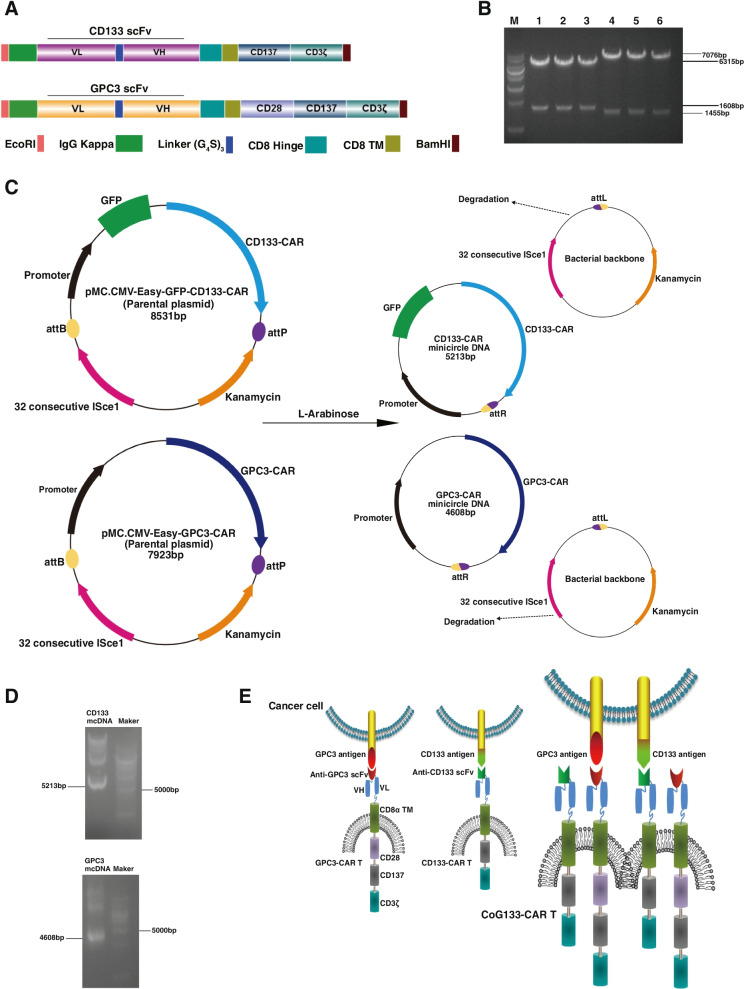
Construction and characterization of two mcDNA vectors and the structure of CoG133-CAR T cells. **A** Schematic representation of CD133-CAR and GPC3-CAR construction. **B** The double restriction enzyme digestion for selecting CAR structures. Lanes 1–3 contain three GPC3-CAR-positive bacterial clones, and lanes 4–6 contain three CD133-CAR-positive bacterial clones. M = molecular weight marker. **C** Schematic diagram showing the generation of the CD133-CAR mcDNA and GPC3-CAR mcDNA. **D** Electrophoretic analysis to detect mcDNA. After L-arabinose induction, CD133-CAR mcDNA and GPC3-CAR mcDNA were digested into fragments of 5179 bp and 4608 bp, respectively. **E** Schematic illustration of the CoG133-CAR T cell structure. CD133-CAR T or GPC3-CAR T cells recognized only one tumor cell surface antigen, but CoG133-CAR T cells exerted a destructive effect on tumors containing CD133 and GPC3 antigens by recognizing one of those antigens

We constructed two nonviral mcDNA vectors encoding GPC3-CAR and CD133-CAR containing the anti-GPC3 and anti-CD133 scFv, respectively. We also constructed a third-generation CAR specific to GPC3 and a second-generation CAR specific to CD133. The anti-GPC3 and anti-CD133 scFv were linked to the intracellular domain of the human CD28 or CD137 and CD3ζ molecules via the human CD8α hinge and CD8α transmembrane regions to enhance costimulatory signaling. To improve the expression efficacy of the chimeric receptor, we added the immunoglobulin kappa light chain (IgG kappa) as the N-terminal leader sequence of anti-GPC3 and anti-CD133 scFv. In addition, to insert the GPC3-CAR and CD133-CAR constructs into the parental plasmid, we added NcoI and EcoRI restriction sites to their 5’- and 3’- ends, respectively (Fig. 1A). We generated two parental minicircle plasmids, pMC.CMV-Easy-GFP-CD133-CAR (8531 bp), with a GFP cassette, and pMC.CMV-Easy-GPC3-CAR (7923 bp), without a GFP cassette (Fig. 1C). On gel electrophoresis, six parental minicircle plasmids were shown to be positive clones as detected by double digestion. Lanes 1–3 held pMC.CMV-Easy-GPC3-CAR containing the GPC3-CAR (1608 bp) and lanes 4–6 held pMC.CMV-Easy-GFP-CD133-CAR containing the CD133-CAR (1455 bp) (Fig. 1B). Two kinds of mcDNA were generated by L-( +)-arabinose-mediated site-specific recombination between attB and attP: CD133-CAR mcDNA (5213 bp) and GPC3-CAR mcDNA (4608 bp) (Fig. 1C and D). We transfected GPC3 mcDNA and CD133 mcDNA to generate GPC3-CAR T and CD133-CAR T cells, respectively, as control groups and simultaneously transfected GPC3-CAR mcDNA and CD133-CAR mcDNA to generate bispecific CoG133-CAR T cells (Fig. 1E).

### Expression of CoG133-CAR in human engineered T cells

We extracted two mcDNA constructs and repeatedly purified them from parental plasmids. GPC3-CAR mcDNA did not encode the GFP gene, and CD133-CAR mcDNA encoded the GFP gene. Then, we simultaneously transfected GPC3-CAR mcDNA and CD133-CAR mcDNA into human T cells via electroporation and generated CoG133-CAR T cells that recognized either the GPC3 or CD133 antigen to induce effector T cell killing of the target tumor cells. Normal T cells without electroporation were established as control cells. Mock T cells were generated by the transfection of the control mcDNA plasmid with the GFP cassette via electroporation; in addition, GPC3-CAR T cells and CD133-CAR T cells were generated by transfection with GPC3-CAR and CD133-CAR mcDNA, respectively, via electroporation.

**Fig. 2:**
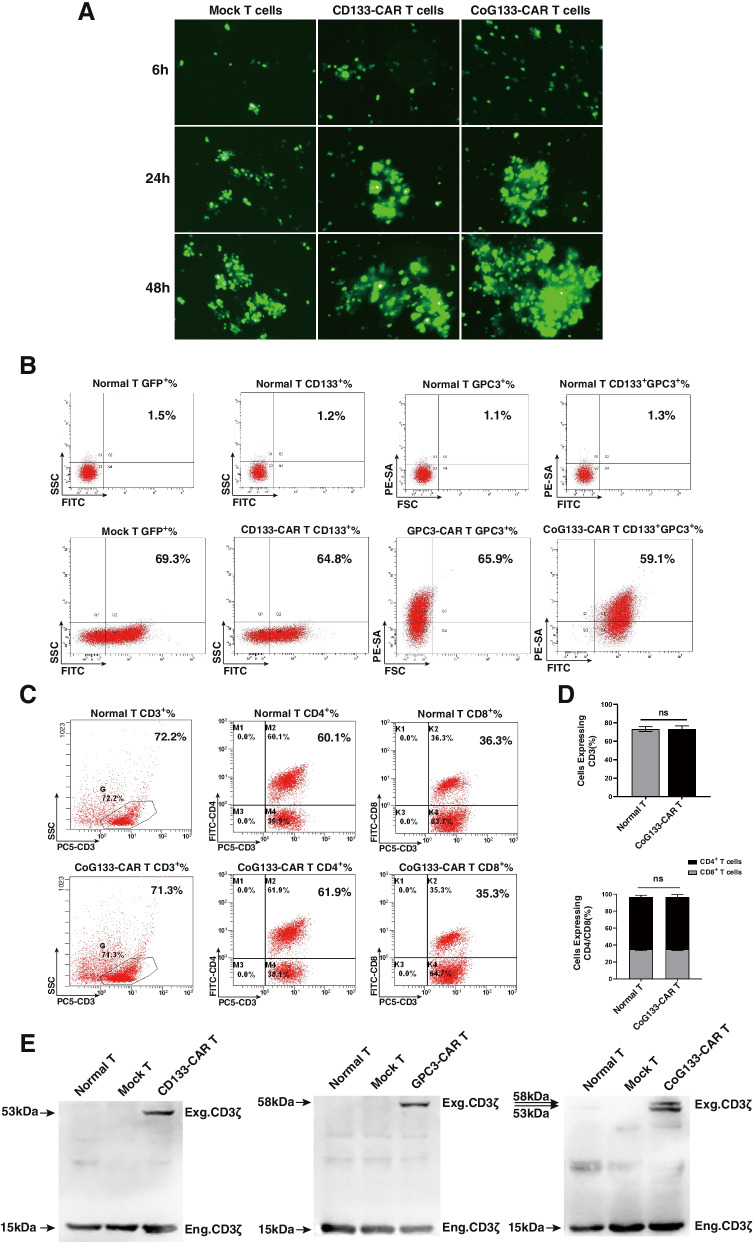
Transfection efficiency and phenotype evaluation of CAR T cells, along with protein expression analysis. **A** Fluorescence microscopy images of human T lymphocytes transfected with the mcDNA or plasmid encoding GFP. The expression of GFP was gradually increased in mock T cells, CD133-CAR T cells and CoG133-CAR T cells 6, 24 and 48 h after electroporation at 400 × magnification. **B** Flow cytometric analysis of CD133-CAR and GPC3-CAR expression in CAR T cells. Seven days after electroporation, the transfection efficiency of mock T, CD133-CAR T, GPC3-CAR T, and CoG133-CAR T cells was 69.3%, 64.8%, 65.9%, and 59.1%, respectively. **C** Flow cytometric analysis showed similar expression levels of CD3, CD4 and CD8 on normal T and CoG133-CAR T cells seven days after electroporation. **D** Graph of the normal T and CoG133-CAR T cell phenotype analysis. Statistics are presented as the means ± SDs. *n* = 3 per group, n.s. not significant. **E** Determination of CAR protein expression after transfection by Western blot analysis. The exogenous CD3ζ protein was detected by chemiluminescence reagents to assess CAR protein expression. The molecular weights of the CD133-CAR and GPC3-CAR proteins were 53 kDa and 58 kDa, and the lysates of CoG133-CAR T cells contained both proteins

Significant green fluorescence was observed by fluorescence microscopy in CAR-engineered T cells encoding the GFP gene 6 h after transfection and constantly increased over the next 24–48 h (Fig. 2A). Seven days after transfection, the expression of CD133-CAR on the cell surface was assessed by determining the GFP expression rate, which was 64.8% and similar to the GFP expression rate in mock T cells. The expression rate of GPC3-CAR on the cell surface was 65.9% and was assessed by staining with an antibody against PE-streptavidin (PE-SA) to detect protein L bound to the GPC3 scFv. The expression of CoG133-CAR was 59.1%, which was assessed by the GPC3/CD133 coexpression rate (Fig. 2B). We evaluated the phenotype of normal T cells and CoG133-CAR T cells by CD3/CD4/CD8 labeling and flow cytometric analysis on day 7. The proportion of CD3^+^ cells was approximately 70%, and the ratio of CD4^+^/CD8^+^ cells was close to 2:1, consistent with the human T cell phenotype under physiological conditions. These results indicated no significant difference in the proportion of CD3^+^, CD4^+^ and CD8^+^ cells between the two groups (Fig. 2C and D). In addition, we used RIPA lysis buffer to extract three kinds of CAR proteins from CAR-engineered T cells, and we then incubated them with an anti-CD3ζ mAb to determine whether GPC3-CAR and CD133-CAR were successfully transfected. In contrast to GPC3-CAR T cells and CD133-CAR T cells, which expressed only a single exogenous CD3ζ protein, CoG133-CAR T cells simultaneously expressed GPC3-scFv-CD28-CD137-CD3ζ(58 kDa) and CD133-scFv-CD137-CD3ζ(53 kDa) fusion proteins, as demonstrated by the successful detection of double exogenous CD3ζ expression. The expression of endogenous CD3ζ protein in T cells was detected in all groups of T cells (Fig. 2E).

### Expression profiles of CD133 and GPC3 in human HCC cell lines and tissues

**Fig. 3:**
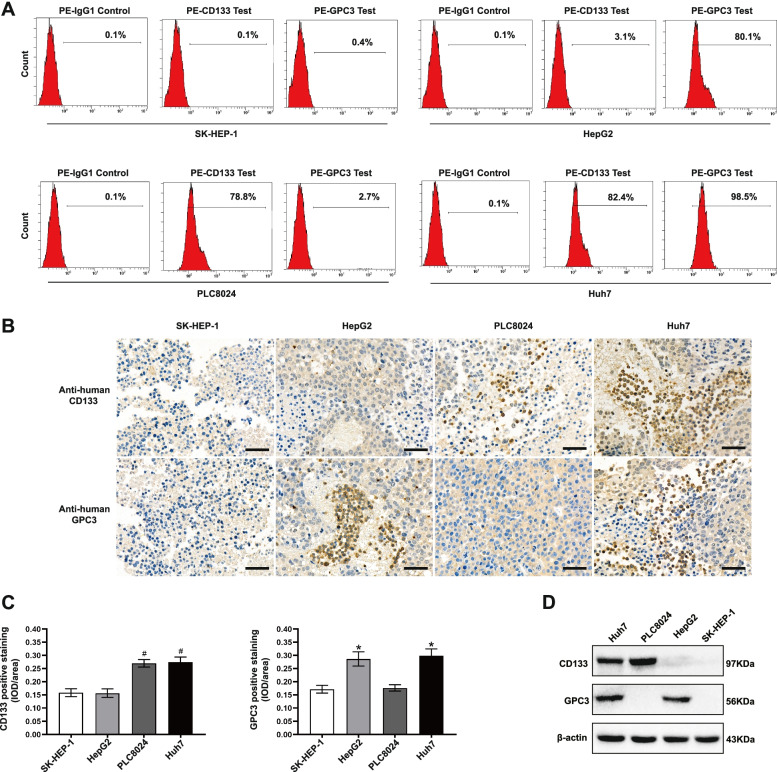
Analysis of CD133 and GPC3 expression in HCC. **A** Flow cytometric analysis of CD133 and GPC3 antigen expression on the surface of cells from 4 human HCC cell lines. Tumor cells were stained with the PE-conjugated anti-IgG1 antibody (isotype control), PE-conjugated anti-CD133 mAb and PE-conjugated anti-GPC3 antibody. **B** Representative immunohistochemical staining images showing CD133 and GPC3 antigen expression in human HCC tissues from NOD/SCID xenograft mice. Scale bar = 50 µm. **C** Semiquantitative IOD analysis of CD133^+^ and GPC3^+^ staining in human HCC cells. Statistics are presented as the means ± SDs. *n* = 3 per group. # *P* < 0.001 vs. the SK-HEP-1 and HepG2 groups. * *P* < 0.001 vs. the SK-HEP-1 and PLC8024 groups. **D** Western blot analysis showing the expression of CD133 and GPC3 proteins in human HCC tissues extracted from NOD/SCID xenograft mice inoculated with SK-HEP-1, HepG2, PLC8024 and Huh7 cells

We stained four types of human HCC cells with PE-conjugated anti-CD133 and PE-conjugated anti-GPC3 mAbs to examine the expression of the CD133 and GPC3 antigens and stained isotype control groups of 4 tumors with a PE-conjugated anti-IgG1 mAb. The expression rates of the CD133 and GPC3 antigens were 0.1% and 0.4%, respectively, in SK-HEP-1 cells; 3.1% and 80.1%, respectively, in HepG2 cells; 78.8% and 2.7%, respectively, in PLC8024 cells; and 82.4% and 98.5%, respectively, in Huh7 cells. We concluded that SK-HEP-1 cells were negative for both antigens, HepG2 cells were positive for the GPC3 antigen, PLC8024 cells were positive for the CD133 antigen and Huh7 cells were positive for both antigens (Fig. 3A). To further demonstrate that the expression of tumor antigens was consistent after the injection of these four human HCC cell lines into NOD/SCID mice and successful modeling, we subjected tumor cells to immunohistochemical staining, and the tumor antigen expression outcomes are shown in optical microscope. HepG2 cells were positively stained with the anti-GPC3 mAb, PLC8024 cells were positively stained with the anti-CD133 mAb, Huh7 cells were positively stained with both antibodies and SK-HEP-1 cells were negative for both antibodies (Fig. 3B). The intensity of CD133 and GPC3 staining in human HCC cells was determined by semiquantitative integrated optical density (IOD) analysis. CD133 antigens were significantly increased in PLC8024 cells and Huh7 cells; GPC3 antigens were significantly increased in HepG2 cells and Huh7 cells (Fig. 3C). We further confirmed the expression of GPC3 and CD133 proteins in human HCC tissues by Western blot analysis. GPC3 protein was expressed in the Huh7 and HepG2 cell lines, and CD133 protein was expressed in the Huh7 and PLC8024 cell lines (Fig. 3D). After verifying the tumor expression profiles by the above methods, we concluded that the SK-HEP-1 cell line was GPC3^−^ and CD133^−^, the HepG2 cell line was GPC3^+^ and CD133^−^, the PLC8024 cell line was GPC3^+^ and CD133^−^, and the Huh7 cell line was GPC3^+^ and CD133^+^.

### Cytotoxicity of CoG133-CAR T cells in vitro

**Fig. 4:**
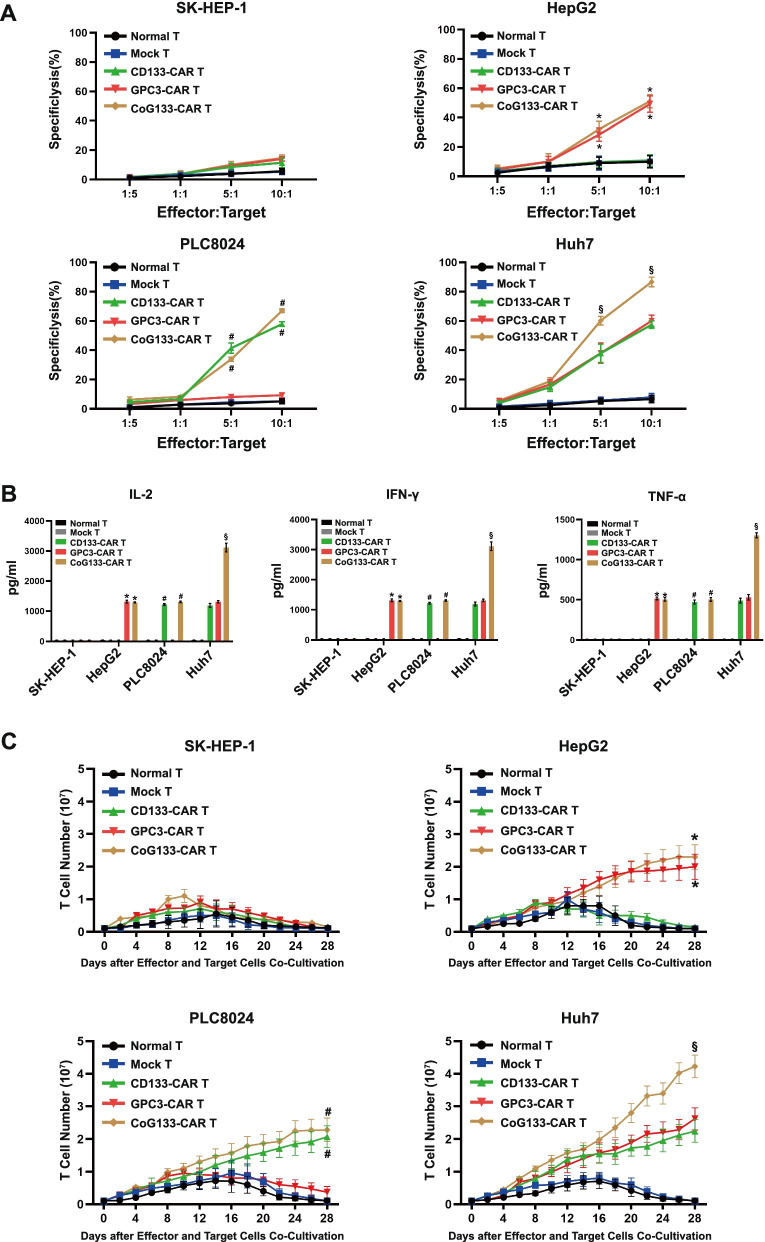
Cytotoxicity activity, cytokine secretion and proliferation of CAR-engineered T cells in vitro. **A** Normal T, mock T and CAR-engineered T cells were co-incubated with human hepatocellular carcinoma cell lines for 18 h at different Effector: Target ratios. **B** ELISA analysis showed the secretion of IL-2, IFN-γ and TNF-α by Normal T, mock T and CAR-engineered T cells which were co-incubated with tumor cells at a 1:1 Effector: Target ratio for 24 h. **C** We prepared 5 × 10^6^ normal T, mock T and CAR-engineered T cells to co-cultivate with human hepatocellular carcinoma cell lines for 28 days. We measured viable T cell numbers every other day to reflect the proliferation of effector cells. All statistics were presented as mean ± SD. *n* = 6 per group, * *P* < 0.001 vs. normal T, mock T and CD133-CAR T groups; # *P* < 0.001 vs. normal T, mock T and GPC3-CAR T groups; § *P* < 0.001 vs. normal T, mock T, CD133 CAR-T and GPC3-CAR T groups

In vitro cytotoxic activity was assessed after overnight coincubation of effector and target cells at distinct ratios. Single-target CAR-engineered T cells such as GPC3-CAR T and CD133-CAR T cells efficiently lysed single-positive tumor cells, and dual-target CAR-engineered T cells such as CoG133-CAR T cells had similar lysis rates against single-positive tumor cells (Fig. 4A). Notably, the lysis rate of double-positive tumor cells showed that the cytotoxic activity of CoG133-CAR T cells was significantly enhanced compared with that of GPC3-CAR T cells and CD133-CAR T cells (Fig. 4A). We used a double-negative SK-HEP-1 cell line as a target cell control group and normal T and mock T cells as effector control groups. The statistical results indicated that the cytotoxic activity was positively correlated with the target-dependent specificity of CAR-engineered T cells.

### Cytokine secretion and proliferation abilities of CoG133-CAR T cells

The intracellular domain of our designed CAR structure originated from CD3ζ and costimulatory signals, and cytokine secretion was enhanced through the resulting synergism. The cytokines secreted by CAR-engineered T cells coincubated with corresponding single-positive tumor cells were increased. Moreover, through interaction with double-positive tumor cells, CoG133-CAR T cells were stimulated to secrete greatly increased levels of cytokines (Fig. 4B). We assessed the proliferative ability of CAR-engineered T cells stimulated by human HCC cells weekly without the addition of exogenous cytokines. The amount of T cells without a CAR structure (normal T and mock T cells) increased moderately at approximately 14 days and decreased after 2 weeks, similar to the effects of coincubation with the 4 tumor cell lines. Single- and double-target CAR T cells coincubated with single-positive tumor cells showed the same proliferative ability; the proliferation of both began to increase after 7 days and expanded approximately 20- to 25-fold after 28 days. After coincubation with the double-positive Huh7 cell line, single-target CAR T cells expanded 20- to 25-fold as above, while dual-target CAR T cells expanded substantially 40-fold (Fig. 4C). We concluded from these statistics that the cytokine secretion and the number of CoG133-CAR T cells were significantly increased by the coincubation with the double-positive tumor cell line.

### Compared with single-target CAR T cells, CoG133-CAR T cells showed significantly improved antitumor activity in vivo against HCC xenografts

**Fig. 5:**
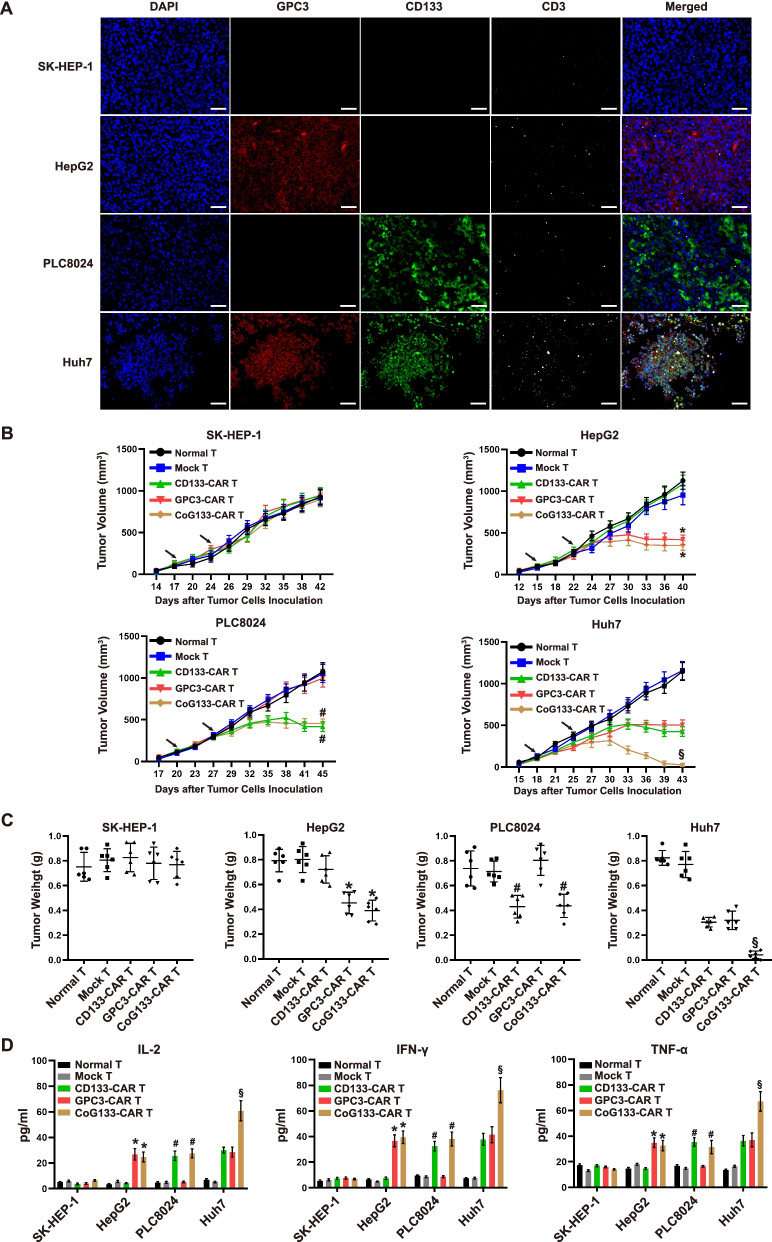
In vivo inhibitory effect of CAR-engineered T cells on tumors. **A** Representative immunofluorescence images of four tumor tissue sections from NOD/SCID xenograft mice injected with CoG133-CAR T cells (sacrificed on day 29 after tumor formation). CD133 is labeled in green, GPC3 is labeled in red and exogenous CD3 is labeled in white; scale bar = 50 μm. **B** We injected effector cells into NOD/SCID xenograft mice on day 0 and day 7 (arrows marked) after the tumor volume was approximately 100 mm^3^ and recorded the tumor volume data. **C** We measured the weight of tumor tissues isolated from the sacrificed mice. **D** ELISA showing cytokine secretion in mouse blood serum seven days after treatment. All statistics are presented as the means ± SDs. *n* = 6 per group. * *P* < 0.001 vs. the normal T, mock T and CD133-CAR T groups; # *P* < 0.001 vs. the normal T, mock T and GPC3-CAR T groups; § *P* < 0.001 vs. the normal T, mock T, CD133-CAR T and GPC3-CAR T groups

Our in vitro results indicated that CoG133-CAR T cells were activated by GPC3^+^CD133^+^ tumor cells and exhibited vigorous antitumor activity against double-positive cell lines. To detect the in vivo efficacy of CoG133-CAR T cells against HCC, we established HCC xenograft mouse models by inoculating 1 × 10^6^ tumor cells from four kinds of cell lines into NOD/SCID mice. We treated the xenograft mice with 1 × 10^7^ effector cells on day 3 and day 10 after tumor formation and then sacrificed them on day 29. Next, we simultaneously detected tumor antigen expression and T cell infiltration by immunofluorescence imaging. GPC3 or CD133 single-positive tumors exhibited a small amount of CD3 white fluorescence after CoG133-CAR T cell treatment. More importantly, Huh7 tumor tissue treated with CoG133-CAR T cells showed a large amount of fluorescence indicating T cell infiltration; in addition, strong positive GPC3 and CD133 fluorescence was simultaneously observed (Fig. 5A). We measured the volume of tumors from xenograft mice and the weight of tumor tissues after the mice were sacrificed (Fig. 5B and C). The tumor size in HepG2 and PLC8024 xenograft mice treated with CoG133-CAR T cells were decreased and did not significantly differ from those in xenograft mice injected with GPC3-CAR T or CD133-CAR T cells. However, the tumor sizes in Huh7 xenograft mice treated with CoG133-CAR T cells were significantly reduced. In vivo cytokine secretion, as detected by ELISA on day 7 showed that the levels of IL-2, IFN-γ and TNF-α in blood serum of GPC3^+^CD133^+^ xenograft mice were the highest among the groups (Fig. 5D). These results indicated that T cells were strongly activated only when CoG133-CAR T cells received integrated costimulatory signals from both the GPC3-targeting antigen and the CD133-targeting antigen, while the activation signals obtained in single-positive tumor-bearing mice were minimal.

### CoG133-CAR T cells exhibited significant growth suppression efficacy in GPC3^+^CD133^+^ tumor xenograft mice

**Fig. 6:**
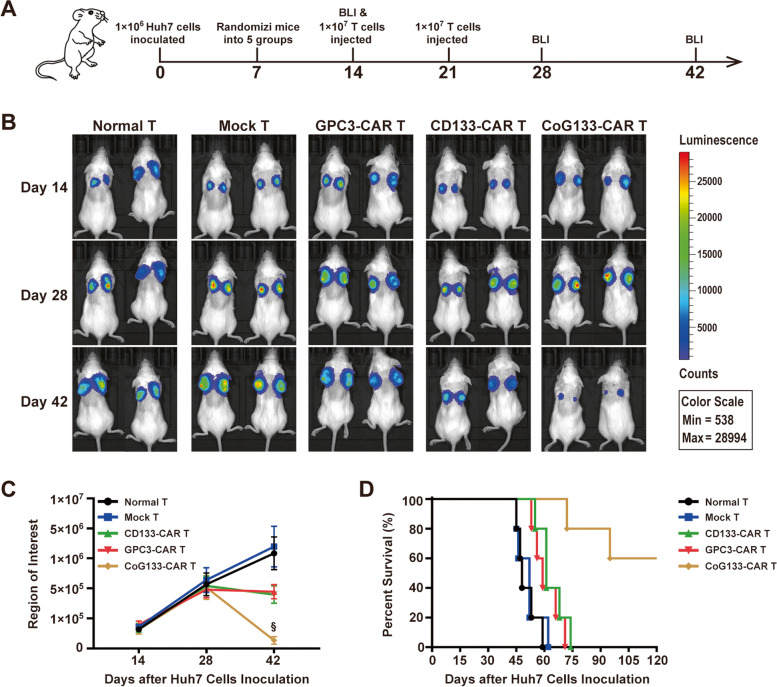
Antitumor efficacy of CoG133-CAR T cells in xenograft mice bearing double-positive tumors. **A** Schematic representation of the in vivo mouse bioluminescence study. **B** The tumor-derived bioluminescence images of mice inoculated with Huh7 cells and treated with normal T and CoG133-CAR T cells. **C** Statistical analysis of the bioluminescence images in the ROI at each time point. Statistics are presented as the means ± SDs. *n* = 5 per group. § *P* < 0.001 vs. the normal T, mock T, CD133-CAR T and GPC3-CAR T groups. **D** Survival curve showed the survival time of Huh7 tumor bearing mice treated with different effector cells. The results were evaluated by the log-rank (Mantel-Cox) test. *n* = 5 per group. § *P* < 0.001 vs. the normal T, mock T, CD133-CAR T and GPC3-CAR T groups

To detect the in vivo suppression efficacy of dual-targeted CAR T cells on GPC3^+^CD133^+^ tumor cells, we established Huh7-NOD/SCID xenograft mouse models by subcutaneously inoculating 1 × 10^6^ Huh7 cells into their dorsal regions on day 0. Subsequently, we randomly divided the mice into 5 groups on day 7. The tumors in xenograft mice grew to 100 mm^3^ on day 14, and bioluminescence images were acquired after the first injection of 1 × 10^7^ effector cells into the mice. We performed a second injection of 1 × 10^7^ effector cells in the mice on day 21 and acquired bioluminescence images on days 28 and 42 (Fig. 6A). The tumor burden was assessed by bioluminescence imaging and revealed that CoG133-CAR T cells induced tumor growth suppression in mice. The tumors disappeared in two of five (40%) xenograft mice, and the other three mice achieved obvious relief by CoG133-CAR T cell injection compared with mice injected with the other effector cells, which exhibited no antitumor efficacy (Fig. 6B). The bioluminescence imaging results were statistically analyzed in a region of interest (ROI) (Fig. 6C). Ultimately, CoG133-CAR T cells induced a significant survival advantage (*n* = 5, *P* < 0.001) (Fig. 6D). In summary, these experimental results indicated that dual-targeted CAR T cells had a potent ability to eradicate Huh7 tumor cells in vivo and prolonged the survival time of Huh7 xenograft mice.

### Detection of the persistence and “off-target” toxicities of CoG133-CAR T cells in vivo

**Fig. 7:**
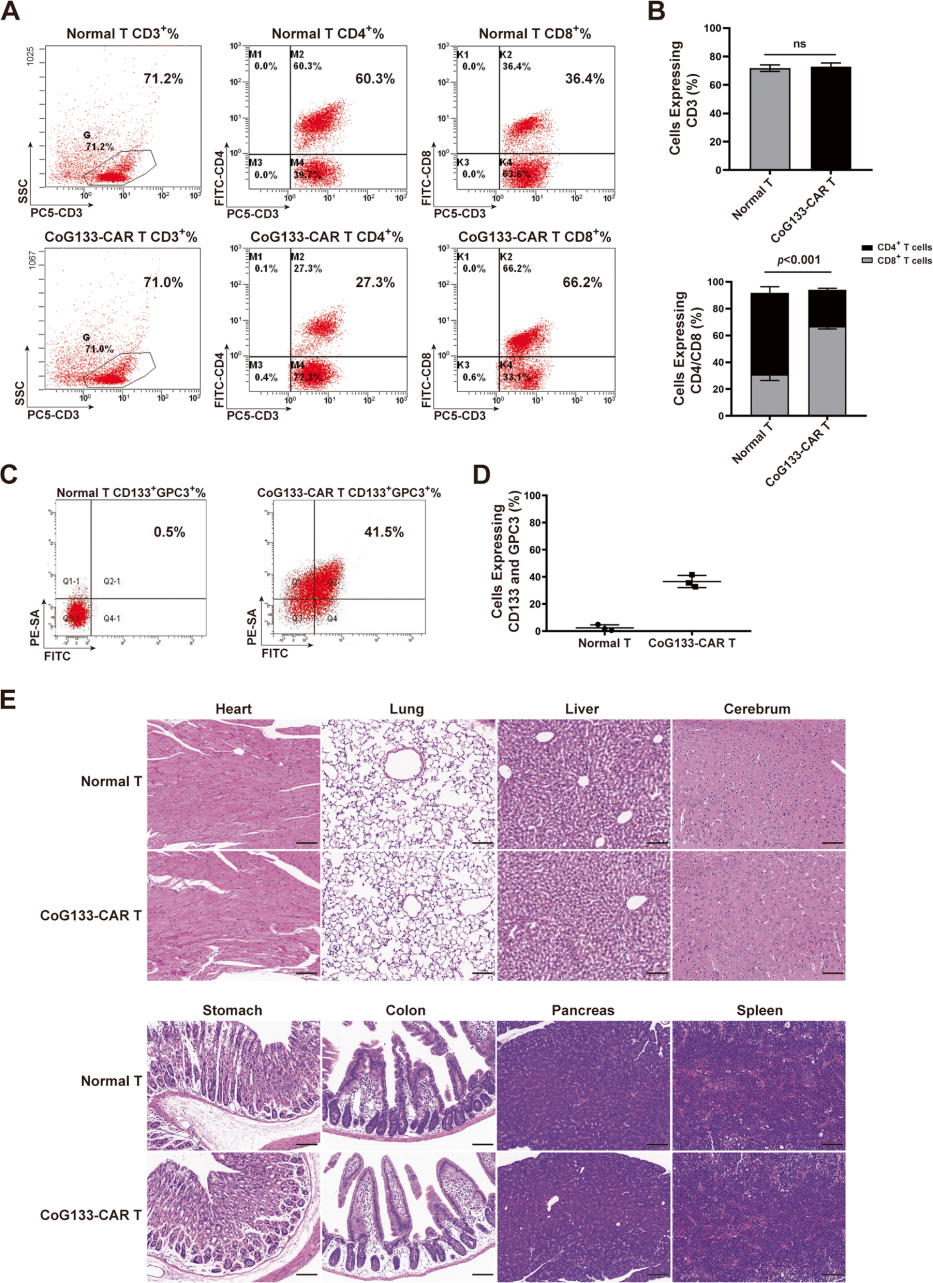
Phenotype, persistence and safety of CoG133-CAR T cells. **A** Flow cytometric analysis showed the phenotype of CAR T and normal T cells from the peripheral blood of Huh7 xenograft mice on day 7. **B** Statistical analysis showed the proportion of CD3^+^, CD4^+^ and CD8^+^ normal T and CoG133-CAR T cells. Statistics are presented as the means ± SDs. *n* = 3 per group, n.s. not significant. *P* < 0.001 vs. the normal T group on CD4/CD8 expression. **C** CD133 and GPC3 coexpression was detected by flow cytometry. **D** Statistical analysis showed the CoG133-CAR T cells expression. Statistics are presented as the means ± SDs. *n* = 3 per group. **E** We harvested murine organ tissues from four groups of tumor xenograft mice sacrificed 28 days after the second injection of effector cells. The tissues were stained with H&E. The histopathological images of the organ tissues from all groups of tumor-bearing mice showed no differences, and images from the Huh7 group are shown as a representative. Scale bar = 100 µm

We detected the phenotypes and the CAR expression of CoG133-CAR T cells from the peripheral blood of Huh7 xenograft mice by flow cytometric analysis on day 7 after the second injection of CoG133-CAR T cells. Normal T cells were used as negative control groups. The proportion of CD4^+^ cells in CoG133-CAR T cells from peripheral blood of mice was significantly reduced compared with that before treatment, but the proportion of CD4^+^ cells in normal T cells did not significantly differ from that before treatment (Figs. 2C, D and 7A, B). More importantly, the CoG133-CAR expression rate was around 40% (Fig. 7C and D). We excised murine organs from sacrificed tumor-bearing mice treated with effector cells and stained them with hematoxylin and eosin (H&E). Histopathological analysis showed no change in the organs from effector cell-treated mice compared to those from untreated mice. The images from Huh7 tumor-bearing mice treated with CoG133-CAR T cells were exhibited only (Fig. 7E).

## Discussion

Immunotherapy has shown broad prospects in cancer treatment [28]. Immune checkpoint inhibitors (anti-PD-1/PD-L1 and anti-CTLA-4 mAbs), dendritic cell (DC)-based vaccines, cytokine-induced killer (CIK) cells, cytotoxic T lymphocytes (CTLs) and CAR-engineered T cells are approved as monotherapies or combination treatments for different types of cancer [29]. In this study, we focused on CAR T cells because of their genetic modification ability, inherent cytotoxic nature and antigen identify characteristic. Our design integrates an mcDNA vector and dual-targeted CAR T cells.

Compared with conventional plasmids, mcDNA is a miniature nonviral DNA vector without a deleterious bacterial backbone that performs well in both gene transfection efficiency and biological safety [30]. We constructed a second-generation CAR structure encoding the CD133 scFv (containing a GFP cassette) and a third-generation CAR structure encoding the GPC3 scFv (excluding a GFP cassette) in parental plasmids. Then, two types of mcDNA vectors were recombined from parental plasmids and simultaneously transfected into primary T cells via electroporation to generate CoG133-CAR T cells. The sizes of the GPC3-CAR mcDNA and CD133-CAR mcDNA were 4608 bp and 5213 bp, respectively (Fig. 1C and D), within the optimal loading range (3000 bp-6000 bp) of mcDNA vectors. Therefore, the simultaneous transfection of two mcDNA-CAR structures into T cells is theoretically reasonable. To illustrate the excellent gene transfection efficiency of the mcDNA vector, strategies to detect CAR expression were adopted, and the CARs exhibited similar expression proportions in both single-specific and bispecific CAR T cells (Fig. 2B).

GPC3 belongs to the heparan sulfate proteoglycan family and is classified as an oncofetal glycoprotein [31]. As an ideal target for adoptive immunotherapy for HCC, GPC3 is overexpressed on the membrane of carcinomatous hepatocytes and negatively detected in normal human tissues and organs [32, 33]. CD133 was first extracted from CD34^+^ hematopoietic stem cells using an anti-AC133 mAb and is specifically expressed in several cancers, including HCC [34, 35]. CD133 is a generally confirmed marker of CSCs with significant impacts on the signal transduction and regulation, proliferation, recurrence and drug resistance of tumors [36, 37]. The expression rates of CD133 and GPC3 in Huh7 cells we confirmed were 82.4% and 98.5%, respectively (Fig. 3A). Differ from the bispecific CAR T cells which contain two targeted antigen-binding sites on one scFv and activate signal transduction pathway only when both TAAs are recognized, our design of bispecific CAR T cells killed tumor cells by binding one of both antigens. The signal activation ability of scFv structures with simultaneous binding to two antigens is much weaker than that of scFv structures with independent binding to two antigens. The CoG133-CAR T cells applied in the present study had two independent scFv antigen-binding sites after GPC3 and CD133 gene sequences were transfected into T cells via mcDNA vectors were generated after the GPC3 and CD133 gene sequences were simultaneously transfected into T cells via mcDNA vectors. Importantly, if we found two antigens highly expressed on a particular type of HCC cell, the corresponding bispecific CAR T cells could exhibit suppression capacity on this HCC cell.

An important reason that malignant tumors are difficult to eradicate is tumor heterogeneity. Tumor heterogeneity originates from the inhomogeneity of the external environment and the randomness of gene mutations and results in the type diversity of cells in the same tumor [38]. CSCs play a critical role in tumor heterogeneity and trigger the self-renewal, multilineage division and sustained growth of tumor cells [39]. As mentioned previously, CD133^+^ tumor cells belong to CSCs, and CD133 antigen is highly expressed in the PLC8024 and Huh7 cell lines that we selected. CD133 localizes to cellular protrusions and guides CD133-targeted T cells to enter CD133^+^ tumor cells for CSCs elimination [40]. Very importantly, these tumor-killing effects overcome the limitation imposed by the tumor microenvironment (TME) by the release of cytokines and CTLs inside the tumor. Two transformations occur in oncology: CSCs can transform into normal cancer cells, and CSCs can transform into normal cells. Both transformations are regulated by the TME [41]. CD133 is a main marker of CSCs with strong cell penetration and GPC3 is a superior HCC marker with high expression.^32^ Since GPC3 antigens are widely present on the surface of HCC cells, the GPC3 branch interacted considerably with GPC3^+^ HCC cells to anchor CoG133-CAR T cells at the tumor and accumulate effector cells around the tumor. The CD133 branch bound CSC-positive HCC cells and activated the intracellular domains of the CAR structures including the CD137 costimulatory molecule and the CD3ζ immunoreceptor tyrosine-based activation motif (ITAM). Then, tumor proliferation and differentiation were inhibited. GPC3 provided extensive adhesion for CoG133-CAR T cells, and CD133 provided profound tumor suppression. Both of these antigens were used in our study to counteract the restrictive effects of the TME.

“Off-target” toxicity is mainly caused by the expression of TAAs in normal tissues. If major human organs express TAAs and are degraded by CAR T cells, life-threatening adverse effects occur in the human body [42]. In this study, T cells were genetically engineered into bispecific CAR T cells containing two tumor antigen-binding sites (CD133 and GPC3) and were used to treat patients with double-positive HCC. The design of corresponding combined sites existed in both effector and target cells, evidently alleviating the “off-target” toxicity, an effect that has been proven in many previous studies [43]. We evaluated vital organs from CoG133-CAR T cell-treated and normal T cell-treated mice via H&E staining. The histopathological images showed that no obvious difference in any vital organ from CoG133-CAR T cell-treated mice (Fig. 7C). Moreover, CoG133-CAR T cells did not have toxicity to normal tissues on mice models.

## Conclusion

Our study indicated that the strategy for mcDNA-mediated CAR-T cells production resulted in excellent transfection efficacy while preventing virus-related adverse effects. The bispecific CAR T cells induced a higher quantity of effector cells to act on double-positive HCC cells and exhibited a CSC-related antitumor ability to destroy the TME. Our work also demonstrated that the design of parallel-connected scFv structures on CoG133-CAR T cells provided precise recognition both in vitro and in vivo. The prolonged survival and tumor reduction were seen in Huh7 xenograft mice treated with CoG133-CAR T cells highlighted the immense prospects of mcDNA vectors and bispecific CAR T cells.

## Supplementary Information

**Additional file 1.**

**Additional file 2.**

**Additional file 3.**

## Data Availability

The nucleotide sequences of CAR structures during the current study are available from the GenBank database (https://www.ncbi.nlm.nih.gov). The corresponding accession numbers are listed as follows, human CD8α hinge and transmembrane domain (nucleotides 412–609, GenBank NM 001,768.6), human CD28 molecule (nucleotides 538–660, GenBank NM 006,139.3), human CD137 molecule (nucleotides 640–765, GenBank NM 001,561.5) and human CD3ζ molecule (nucleotides 154–492, GenBank NM 198,253.2).

## Abbreviations

HCC: Hepatocellular carcinoma
CSC: Cancer stem cell
GPC3: Glypican-3
CAR: Chimeric antigen receptor
mc: Minicircle
HBV: Hepatitis B virus
HCV: Hepatitis C virus
CR: Clinical remission
scFv: Single-chain variable fragment
TAA: Tumor-associated antigen
HLA: Human leukocyte antigen
PBMC: Peripheral blood mononuclear cell
DC: Dendritic cell
CIK: Cytokine-induced killer
CTL: Cytotoxic T lymphocyte
TME: Tumor microenvironment
ITAM: Immunoreceptor tyrosine-based activation motif
