# Supplementary figures and images for: Nonviral mcDNA-mediated bispecific CAR T cells kill tumor cells in an experimental mouse model of hepatocellular carcinoma
Source: BMC Cancer. 2022 Jul 25;22:814. doi: 10.1186/s12885-022-09861-1 (PMC9310485; doi:10.1186/s12885-022-09861-1)

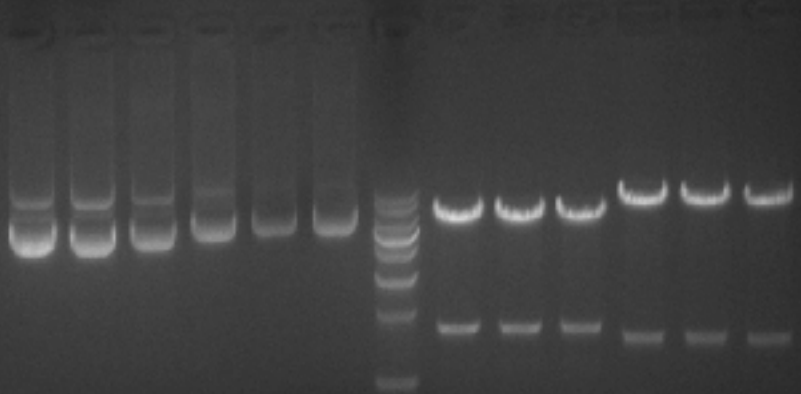

Supplement: Supplementary file 1 — Additional file 1. [file 12885_2022_9861_MOESM1_ESM.zip › Supplementary Figure1B.pdf]

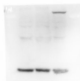

Supplement: Supplementary file 2 — Additional file 2. [file 12885_2022_9861_MOESM2_ESM.zip › Supplementary Figure2E-1-1.pdf]

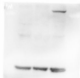

Supplement: Supplementary file 2 — Additional file 2. [file 12885_2022_9861_MOESM2_ESM.zip › Supplementary Figure2E-1-2.pdf]

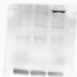

Supplement: Supplementary file 2 — Additional file 2. [file 12885_2022_9861_MOESM2_ESM.zip › Supplementary Figure2E-2-1.pdf]

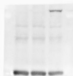

Supplement: Supplementary file 2 — Additional file 2. [file 12885_2022_9861_MOESM2_ESM.zip › Supplementary Figure2E-2-2.pdf]

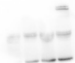

Supplement: Supplementary file 2 — Additional file 2. [file 12885_2022_9861_MOESM2_ESM.zip › Supplementary Figure2E-3-1.pdf]

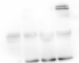

Supplement: Supplementary file 2 — Additional file 2. [file 12885_2022_9861_MOESM2_ESM.zip › Supplementary Figure2E-3-2.pdf]

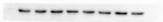

Supplement: Supplementary file 3 — Additional file 3. [file 12885_2022_9861_MOESM3_ESM.zip › Supplementary Figure3D-actin.pdf]

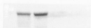

Supplement: Supplementary file 3 — Additional file 3. [file 12885_2022_9861_MOESM3_ESM.zip › Supplementary Figure3D-CD133.pdf]

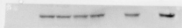

Supplement: Supplementary file 3 — Additional file 3. [file 12885_2022_9861_MOESM3_ESM.zip › Supplementary Figure3D-GPC3.pdf]
