# Supplementary material for: Nonviral mcDNA-mediated bispecific CAR T cells kill tumor cells in an experimental mouse model of hepatocellular carcinoma
Source: BMC Cancer. 2022 Jul 25;22:814. doi: 10.1186/s12885-022-09861-1 (PMC9310485; doi:10.1186/s12885-022-09861-1)

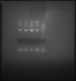

Supplement: Supplementary file 1 — Additional file 1. [file Supplementary Figure1D-1.pdf › Supplementary Figure1D-1.pdf]
